# Dynamic response of cerebral blood flow to insulin-induced hypoglycemia

**DOI:** 10.1038/s41598-020-77626-6

**Published:** 2020-12-04

**Authors:** Ruth McManus, Seva Ioussoufovitch, Elizabeth Froats, Keith St Lawrence, Stan Van Uum, Mamadou Diop

**Affiliations:** 1 St. Joseph’s Health Care, London, ON N6A 4V2 Canada; 2 Department of Biomedical Engineering, Western University, London, ON N6A 5C1 Canada; 3 Department of Medical Biophysics, Western University, London, ON N6A 5C1 Canada

**Keywords:** Endocrinology, Optics and photonics

## Abstract

The dynamics of cerebral blood flow (CBF) at the onset of hypoglycemia may play a key role in hypoglycemia unawareness; however, there is currently a paucity of techniques that can monitor adult CBF with high temporal resolution. Herein, we investigated the use of diffuse correlation spectroscopy (DCS) to monitor the dynamics of CBF during insulin-induced hypoglycemia in adults. Plasma glucose concentrations, cortisol levels, and changes in CBF were measured before and during hypoglycemia in 8 healthy subjects. Cerebral blood flow increased by 42% following insulin injection with a delay of 17 ± 10 min, while the onset of hypoglycemia symptoms was delayed by 24 ± 11 min. The findings suggest that the onset of CBF increments precedes the appearance of hypoglycemia symptoms in nondiabetic subjects with normal awareness to hypoglycemia, and DCS could be a valuable tool for investigating the role of CBF in hypoglycemia unawareness.

## Introduction

Hypoglycemia, defined as plasma glucose concentration levels below 3 mM^1^, is a rare occurrence in healthy people but a common complication of diabetes therapy. Notably, people with Type 1 diabetes mellitus or insulin‐treated Type 2 diabetes mellitus experience up to 94 and 36 hypoglycemic episodes every year, respectively, potentially including severe and life-threatening events^2^. This often leads to fear of hypoglycemia and, consequently, exaggerated avoidance behaviour resulting in poor glycemic control^2,3^. Fear of severe hypoglycemia represents a major obstacle to optimum therapy and disease management in diabetes mellitus.

Hypoglycemia is typically accompanied by neurogenic symptoms (e.g., sweating, pallor, anxiety, hunger, and tremor) that alert the body to the low glucose level before the appearance of more serious neuroglycopenic symptoms such as confusion, blurred vision, seizures, and loss of consciousness^4^. However, recurrent hypoglycemia can lead to the appearance of neuroglycopenic symptoms without the normal preceding neurogenic warning symptoms ‒ a condition known as hypoglycemia unawareness. Hypoglycemia unawareness is a serious condition and is associated with significant morbidity and mortality^5^.

Glucose is the only source of energy for neural tissue under normal circumstances, and because the brain cannot synthesize glucose and has very limited storage of energy substrate, continuous supply of glucose is essential for normal cerebral function; any prolonged disruption in supply can have devastating consequences on the brain^6,7^. Not surprisingly, hypoglycemia has been associated with increased cerebral blood flow (CBF)—presumably to increase substrate delivery and compensate for the lower level of blood glucose^8,9^. In healthy subjects, hypoglycemia causes increased CBF in specific regions of the brain such as the prefrontal cortex, thalamus, and globus pallidum^10^. However, such spatial redistribution of cerebral perfusion was not observed in diabetes patients with impaired awareness of hypoglycemia^11^. Instead, these patients experienced a global increase in CBF, which is believed to blunt the symptomatic response to hypoglycemia since increased CBF at the onset of systemic hypoglycemia is associated with diminished perception of low blood glucose levels^9,11^. This hypothesis is supported by the findings that acetazolamide-induced increase in CBF reduces the severity of hypoglycemia symptoms^9^, and caffeine-induced reduction in CBF increases the severity of hypoglycemia symptoms^12^. Together, these studies strongly suggest that the dynamic relationship between CBF and blood glucose concentration plays a key role in hypoglycemia awareness.

The classic approach to studying the effects of hypoglycemia on cerebral perfusion in humans is to use glycemic clamp to obtain a steady-state blood glucose level, and map the spatial changes in CBF with medical imaging modalities such magnetic resonance imaging (MRI)^11,13^, positron emission tomography (PET)^10^, and single photon emission computed tomography (SPECT)^14^. Although this approach has provided valuable insights into the spatial redistribution of cerebral perfusion due to low blood glucose, the dynamics of CBF during hypoglycemia remains poorly understood because of the inherent low temporal resolution of the method, and limitations of current perfusion modalities such as availability (MRI) and use of ionizing radiations (PET and SPECT). Herein, we investigate the use of diffuse correlation spectroscopy to monitor the *dynamic* response of CBF to acute hypoglycemia in adult subjects.

Diffuse correlation spectroscopy (DCS) is a portable optical technique that can measure the dynamics of light scatterers in turbid media, including living tissue. The technique uses a long coherence length light source so that, despite their different pathlengths, photons that exit the medium still interfere because the coherence length of the DCS light source is always longer that the largest difference between the pathlengths of the photons. The decorrelation of the light interference at the surface of the medium is directly related to the movement of the light scatterers inside the medium. In living tissue, this decorrelation is mainly due to movement of red blood cells and hence, the DCS correlation curve decays faster when the red blood cells move faster (i.e., when blood flow increases). Conversely, the DCS correlation curve decay slower when blood flow is slower. Many studies, including ours^15–18^, have shown that *changes* in the macroscopic diffusion coefficient that characterizes the DCS correlation curve strongly agree with *changes* in tissue (e.g., brain) perfusion measured by Positron Emission Tomography^19^ and Arterial Spin Labelling MRI^20,21^, and were validated against fluorescence microspheres perfusion measurements^22,23^. Further, DCS is safe and can be deployed in the clinic with minimum interference with patient care to monitor CBF^24,25^, muscle blood flow^26,27^, and breast perfusion^28^. It is, however, noteworthy that although *changes* in the diffusion coefficient measured by DCS correlate strongly with *changes* in tissue perfusion, current DCS techniques do not measure absolute tissue blood flow.

In the current study, we recruited adult subjects with normal awareness of hypoglycemia, who were undergoing an insulin-induced hypoglycemic challenge as part of their clinical care. We hypothesized that hypoglycemia would cause increased blood flow in the prefrontal cortex and this could be detected by placing DCS probes on the scalp of the subjects’ forehead.

## Methods

### Instrumentation

**Figure 1:**
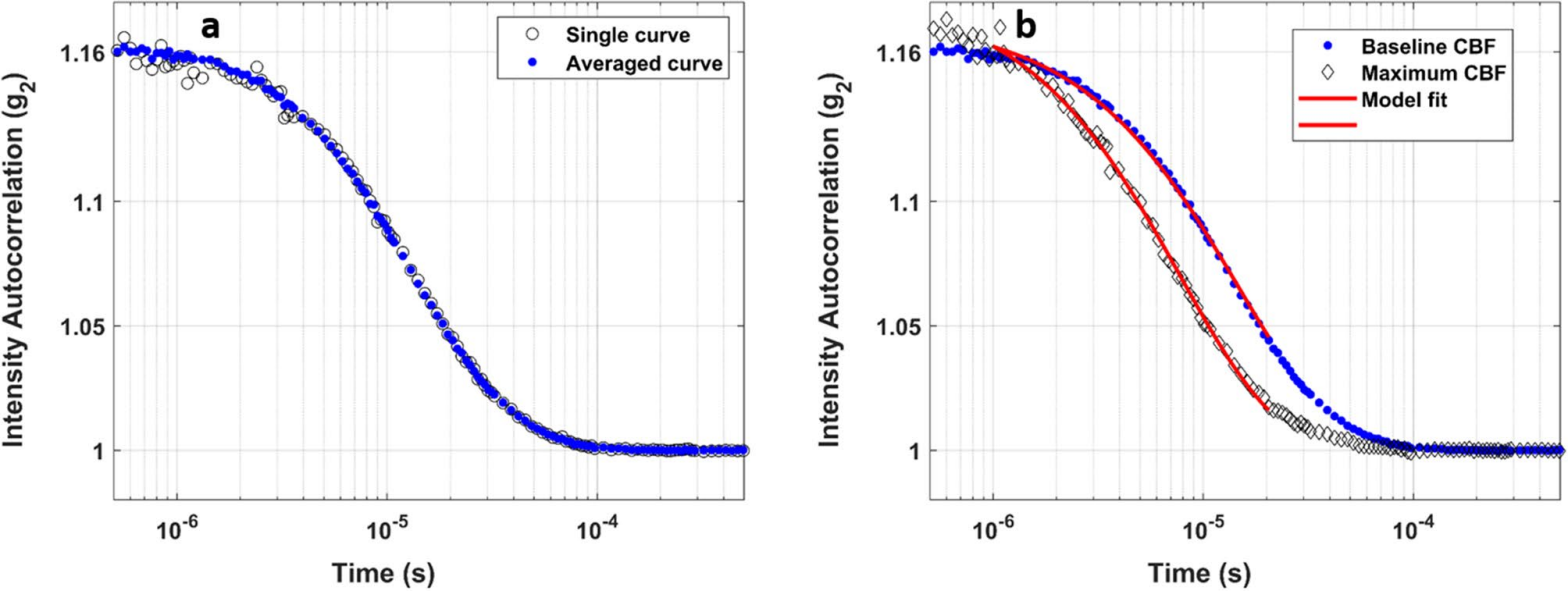
(**a**) Typical DCS curve (i.e., intensity autocorrelation curve) measured by one of the six channels of the detection probe (open grey circles) and the higher SNR curve (blue dots) obtained by averaging the autocorrelation curves measured by all channels. (**b**) Examples of DCS curves measured in one subject at baseline (blue dots) and at the highest cerebral blood flow index (open grey diamonds). The solid red curves are the fit of the theoretical model to the data, using a fitting range from 1 to 20 µs, to estimate the diffusion coefficients (αD_B_).

The DCS device was built in-house using a continuous-wave laser emitting at 785 nm (DL785-100-S, CrystaLaser, Nevada, US). The maximum output power of the laser was 70 mW, and its coherence length was greater than 5 m. The laser beam was first attenuated by a variable neutral density filter (NDC-50-4M, Thorlabs, New Jersey, US), then coupled into the emission probe (single multimode optical fiber: N.A. = 0.22, core = 400 µm, outer diameter of the sheathing = 4.7 mm; Fiberoptics Technology, Connecticut, US) to guide the light to the subject’s forehead. Multiply scattered photons that were reemitted from the head were collected using the detection probe (6 single-mode SMF-28e + fibers, bundled together at the subject side: N.A. = 0.14, length = 4 m, core = 8.2 µm, single-mode cut-off wavelength at 1260 nm). The higher-order modes of the detection fibers, which guide few-modes at the laser emission wavelength, were converted into non-propagating modes by wrapping the fibers into individual 5-cm coils^29^. The output of each detection fiber was coupled to one of the input-channels of two sets of four single photon counting modules (SPCM-AQ4C, Excelitas Canada Inc)^30^. The outputs of the SPCM modules were sent to a photon correlator board (DPC-230, Beker & Hickl) that computed the intensity autocorrelation function for each channel. The sampling rate of the DCS was set to 60 s; that is, each detection channel provided one autocorrelation curve every minute. The autocorrelation curves from all six channels were averaged to yield a single curve, with improved signal-to-noise ratio (Fig. 1a); this curve contained information about the dynamics of the moving light scatterers (mainly red blood cells) in the brain region probed by the detected photons.

### Subjects and experimental protocol

We recruited nondiabetic volunteers aged 18 years and older who were undergoing an insulin-induced hypoglycemia test because of clinical suspicion of inadequate cortisol reserve. Controlled hypoglycemia was intentionally induced as part of standard-of-care to stimulate the hypothalamic/pituitary/adrenal axis. The study was conducted in the morning after an overnight fast. Subjects were seated comfortably with the DCS probe-holder (2 cm × 5 cm; 3D printed using NinjaFlex) positioned on the scalp of the forehead and secured by an adjustable headband. Thereafter, the emission and detection probes were placed in the probe-holder to assess the frontal cortex. This choice was guided by previous findings that CBF increases in the frontal cortex during hypoglycemia^14,31^, and the accessibility of the cerebral cortex to the DCS light when probes are positioned on the scalp. More specifically, similar to other continuous-wave near-infrared spectroscopy techniques, the expected depth of penetration of the DCS light is equal to the square root of the distance between the emission and the detection probes^32,33^. Since probes were placed 3 cm apart, the expected depth of penetration of the DCS light is about 1.4 cm; thus, the DCS will probe the scalp, skull, and cortical region beneath the probes but will not to be sensitive to changes in subcortical regions. Furthermore, plasma blood glucose (BG) were measured at baseline, followed by a bolus injection of Regular Insulin (typically 0.1 units/kg). Plasma BG and cortisol levels were sampled every 15 min, during the first 45 min, and every 30 min thereafter for 2 h while CBF was continuously monitored with the DCS device for 60–90 min. Since the delay between the insulin injection and the start of the DCS measurements was not the same for all subjects, the time of insulin injection was taken as temporal reference for each subject. Subjects were observed and asked to rate their symptoms of hypoglycemia throughout the test. Subjects were also asked to report the onset of low glucose symptoms. The study was conducted in the outpatient endocrinology clinic at St Joseph’s Health Care, London (Ontario, Canada) and written informed consent was obtained from all subjects. The study protocols/procedures were approved by the Western University Health Sciences Research Ethics Board, which adheres to the guidelines of the Tri-Council Policy Statement (TCPS), Ethical Conduct for Research Involving Humans.

### Data analysis

The normalized intensity autocorrelation function generated by the DCS device can be described by^34^:

1 $$g_{2} \left( {\rho ,\tau } \right) \propto \frac{<{I\left( {\rho ,t} \right)I\left( {\rho ,t + \tau } \right)}>}{<{I\left( {\rho ,t} \right)}>^{2}},$$

where $I\left( {\rho ,\tau } \right)$ and $I\left( {\rho ,t + \tau } \right)$ are the light intensities measured by the detector at a given time $t$, and $\tau$ units of time later; $\rho$ is distance between the emission and detection probes (i.e., the source-detector separation); $<\ldots>$ denotes temporal averaging.

However, the theoretical model of light scattering by the moving red blood cells does not yield $g_{2} \left( {\rho ,\tau } \right)$ but instead, provides the electric field autocorrelation function *G*_*1*_(*ρ,τ*). Nevertheless, $g_{2} \left( {\rho ,\tau } \right)$ can be expressed as a function of *G*_*1*_(*ρ,τ*) via the Siegert relation^35^:

2 $$g_{2} \left( {\rho ,\tau } \right) = 1 + \beta \frac{{\left| {G_{1} \left( {\rho ,\tau } \right)} \right|^{2} }}{<{I\left( \rho \right)}>^{2}}$$

Here *β* is the coherence factor and $<I\left( \rho \right)>$ represents the average intensity measured by the detector.

It has been shown that in highly scattering, low absorbing media such as brain tissue, *G*_*1*_(*ρ,τ*) is solution to the diffusion equation when the source-detector separations is large (i.e., *ρ* > 3/*µ*_*s*_*'*; *µ*_*s*_*'* is the reduced scattering coefficient of the tissue)^36^. In the current study, the DCS data were analyzed using the analytical solution to the diffusion equation for a semi-infinite homogeneous medium with extrapolated-zero boundary conditions^37^:

3 $$G_{1} (\rho ,\tau ) = \frac{{3\mu^{\prime}_{s} }}{4\pi }\left( {\frac{{\exp ( - k_{D} r_{1} )}}{{r_{1} }} - \frac{{\exp ( - k_{D} r_{2} )}}{{r_{2} }}} \right)$$

**Figure 2:**
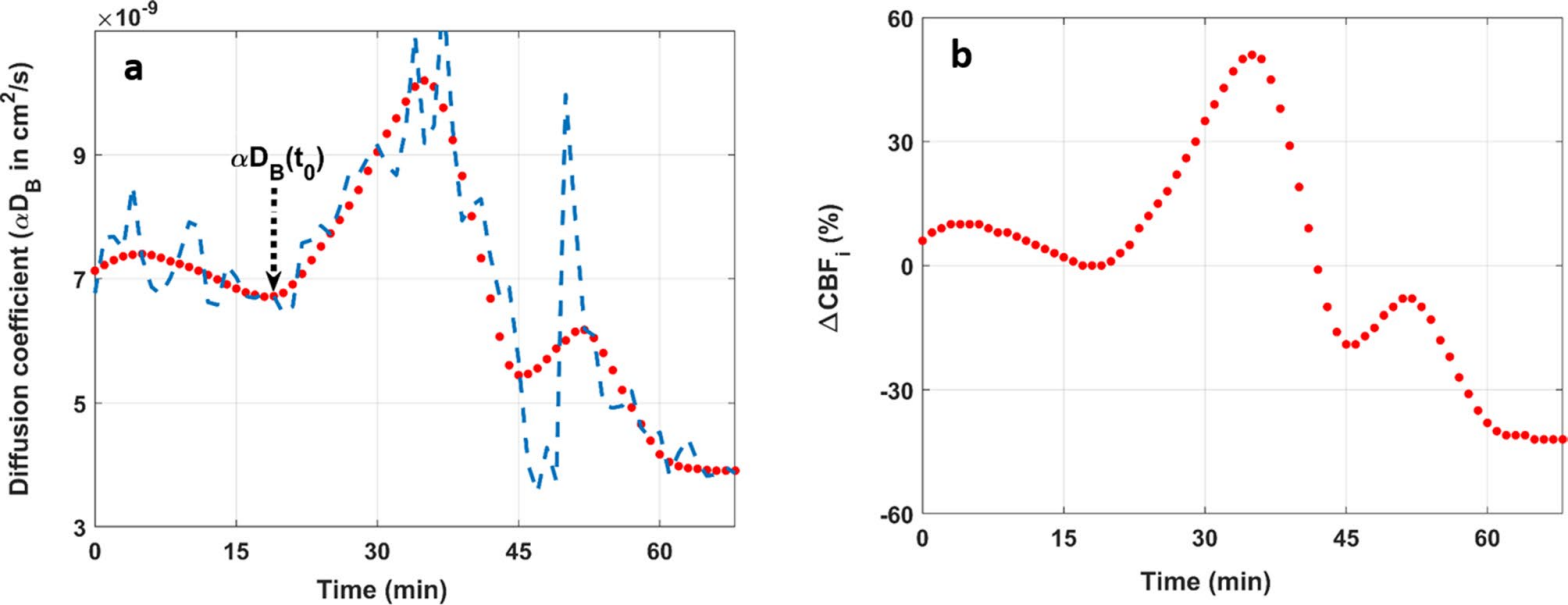
(**a**) Dashed blue curve is an example of time-dependent diffusion coefficient, αD_B_(t), obtained by fitting the autocorrelation curves measured over time in a single subject. Note that insulin was injected at t = 0. The red curve in (**a**) was obtained by denoising the data of the blue curve. αD_B_(t_0_) is the reference diffusion coefficient and corresponds to the onset of the rise of αD_B_(t). The curve in (**b**) was obtained by applying Eq. (4) to the denoised αD_B_(t) data (i.e., the data of the red curve in a).

Here, $k_{D}^{2} = 3{\mu^{\prime}_{s}} {\mu_{a}} + 6\mu^{\prime 2}_{s} {k_{0}^{2}} \alpha {D_{B}} \tau$ (for Brownian dynamics^29^); $r_{1} = \sqrt {\rho^{2} + z_{0}^{2} } ;r_{2} = \sqrt {\rho^{2} + (z_{0} + 2z_{b} )^{2} }$; *ρ* is the source-detector separation; *z*_*b*_ is the distance above the surface of the head where the fluence vanishes; *z*_*0*_ is the effective depth of the source; *µ*_*a*_ is the tissue absorption coefficient; and *α* is the probability of light scattering by a red blood cell^38^. Each measured $g_{2} \left( {\rho ,\tau } \right)$ curve was fit with the above analytical model and Eq. (2), using the known source-detector separation and assuming typical brain optical properties: *µ*_*s*_*'* = 10 cm^−1^ and *µ*_*a*_ = 0.2 cm^−1^^39^. This procedure yields estimates of *β* and the scaled diffusion coefficient (*αD*_*B*_), which we will refer to as the diffusion coefficient for the remaining of the manuscript. The fitting range of the measured $g_{2} \left( {\rho ,\tau } \right)$ curves was between 1 µs and 20 µs (Fig. 1b). The lower limit was chosen to reduce the influence of noise at early correlation times, while the upper limit was set to not overweight contributions from the tail of the autocorrelation curve and to reduce contributions from photons with short pathlengths, which have mainly probed extracerebral tissues. To reduce noise, while preserving the general trend, the time-dependent diffusion coefficients (i.e., $\alpha D_{B} \left( t \right)$) obtain from each subject were denoised using a wavelet denoising approach^40^ (see Fig. 2a for illustration). Thereafter, changes in cerebral blood flow index ($CBF_{i}$) were estimated as follows^16^:

4 $$\Delta CBF_{i} \left( t \right) = 100 \times \frac{{\alpha D_{B} \left( t \right) - \alpha D_{B} \left( {t_{0} } \right)}}{{\alpha D_{B} \left( {t_{0} } \right)}}$$

$\alpha D_{B} \left( t \right)$ is the time-dependent diffusion coefficient (in units of cm^2^/s) measured by the DCS at any given time $t$; $\alpha D_{B} \left( {t_{0} } \right)$ is the reference diffusion coefficient and was chosen to coincide with the initial rise of $\alpha D_{B} \left( t \right)$ as illustrated in Fig. 2a; $\Delta CBF_{i} \left( t \right)$ is the percent change in cerebral blood flow index at any given time, compared to baseline (i.e., at time $t_{0}$; see Fig. 2b for illustration). Note that we are referring to the changes in the DCS diffusion coefficient as changes in cerebral blood flow *index* (not cerebral perfusion); however, it has been shown that changes computed with Eq. (4) correlate strongly with changes in tissue blood flow measured by PET and MRI perfusion methods^19–21^.

**Figure 3:**
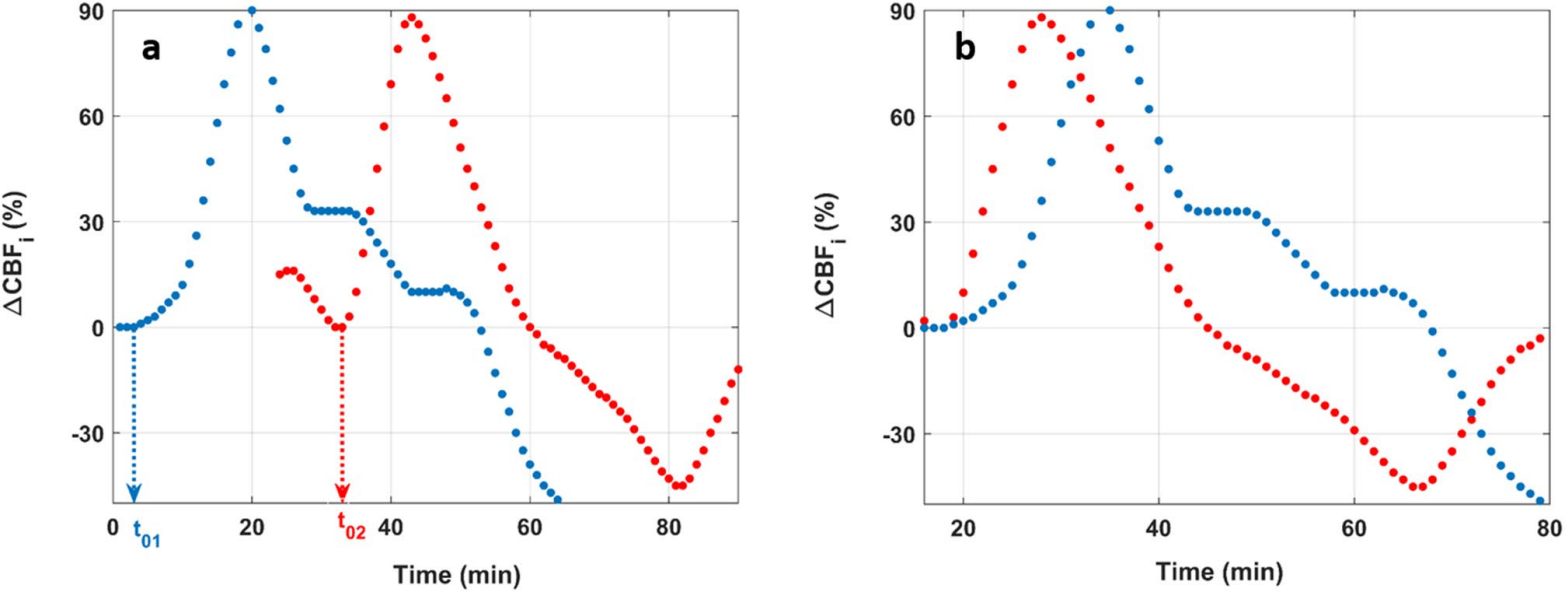
(**a**) Examples of changes in CBFi from two subjects; the time of the insulin bolus was chosen as the origin of time (i.e., t = 0). The curves in (**b**) are the same as those in (a) but were shifted so that t_01_ and t_02_, the time points of the initial rise of CBFi, coincide.

Furthermore, since we expected $t_{0}$ (i.e., the onset of the rise of $CBF_{i}$) to be different between individuals, $\Delta CBF_{i} \left( t \right)$ from each subject was temporally shifted so that all $t_{0}$ coincide (Fig. 3). This temporal shift was necessary to account for physiological heterogeneity such as individual response to the insulin injection. Once the rise of all $\Delta CBF_{i} \left( t \right)$ curves were temporally aligned, the mean (standard error; SE) $\Delta CBF_{i}$ at each time $t$ was computed as the average of $\Delta CBF_{i} \left( t \right)$ from all subjects at that time.

### Statistical analysis

Statistical analysis was conducted using SPSS Statistics 25 and power analysis was performed using G*Power software. For the cortisol measurements, 2 out of 8 subjects’ measurements (subject 2 and subject 6) were missing at the 0-min timepoint, and 2 subjects’ measurements were missing at the 15- and 90-min timepoints (subject 6 and subject 1, respectively). For the blood glucose measurements, subject 6 was missing measurements at the 0- and 15-min timepoints. Multiple imputation was used to account for missing data: variables were imputed under the fully conditional specification using the default settings in SPSS. Ten multiply imputed datasets were created; pooled estimates of mean cortisol and blood glucose values for each timepoint provided by SPSS were used to perform subsequent statistical analysis.

A one-way repeated measure analysis of variance (ANOVA) was conducted on blood glucose and cortisol data with time as the within-subjects variable. Normality of the data was confirmed visually using Q-Q plots generated in SPSS, and sphericity of the data was confirmed using Mauchly’s test of sphericity. Thus, it was determined that no assumptions inherent to ANOVA were violated. Upon discovering a significant effect, differences between timepoints were uncovered using a post-hoc Tukey’s honest significant difference test to account for multiple comparisons. The power of the findings was calculated using a post-hoc test in G*Power and an effect size equal to the partial *η*^2^ value determined using ANOVA.

## Results

We recruited eight non-diabetic subjects (6 females) undergoing an insulin-induced hypoglycemia test for the assessment of pituitary function. The age (mean ± SD) of the subjects was 42.5 ± 14.2 years and their average weight (mean ± SD) was 92.9 ± 30.8 kg. Two subjects (subject 1 and 6) received a second dose of insulin because the first injection did not achieve clinically-relevant hypoglycemia. Measurements were completed in all eight subjects and the DCS was very well tolerated with no adverse events arising from the optical measurements.

Figure 1a displays an example of DCS curve measured by one detector (open grey circles) and the higher signal-to-noise ratio (SNR) curve obtained by averaging the autocorrelation curves measured by all six channels of the detection probe. The SNR improvement is particularly noticeable at early correlation times (e.g., lower than 4 µs) for which the autocorrelation curves from individual channels tend to be noisier. Figure 1b shows a high SNR DCS curve measured at baseline (blues dots) and a faster (left shift) decaying autocorrelation curve measured at the highest ${CBF}_{i}$ in one subject. The solid red curves are the fit of the theoretical model Eq. (2) to the measured autocorrelation data to quantify the diffusion coefficients, which are then used to compute the changes in ${CBF}_{i}$ using Eq. (4).

Figure 2a shows the time-dependant diffusion coefficient, $\alpha {D}_{B}\left(t\right)$, measured in one subject. The dashed blue curve represents the diffusion coefficients obtained by fitting the higher SNR autocorrelation curves measured at each time (e.g., Fig. 1b). The smooth red curve in Fig. 2a was obtained by denoising the raw $\alpha {D}_{B}\left(t\right)$ data. The diffusion coefficient at the initial rise is shown as $\alpha {D}_{B}\left({t}_{0}\right)$ in Fig. 2a and was used as reference to compute the changes in ${CBF}_{i}$ from the denoised $\alpha {D}_{B}\left(t\right)$ data; the resulting changes in ${CBF}_{i}$ are shown in Fig. 2b.

Figure 3 illustrates the temporal shifting of the $\Delta CBF_{i} \left( t \right)$ curves so that their initial rises coincide. Note that the time of insulin injection was chosen as reference for the x-axis and the initial time of each curve correspond to the start of the DCS acquisition with respect to the time of the insulin bolus. For example, the blue curve in Fig. 3a shows that for this subject the DCS acquisition was started at the same time as the insulin injection and for the red curve, the DCS acquisition was started 24 min after the insulin bolus. Figure 3b shows the shifting of the curves in Fig. 3a so that the onset of the rise of their ${CBF}_{i}$ overlap (i.e., t_01_ and t_02_ coincide). As a result, the time axis in Fig. 3b is the average of the shifted time. For example, since t_01_ = 3 min and t_02_ = 33 min, the initial rise of the shifted $\Delta CBF_{i} \left( t \right)$ curves in Fig. 3b is 18 min, which is the average of t_01_ and t_02_.

**Figure 4:**
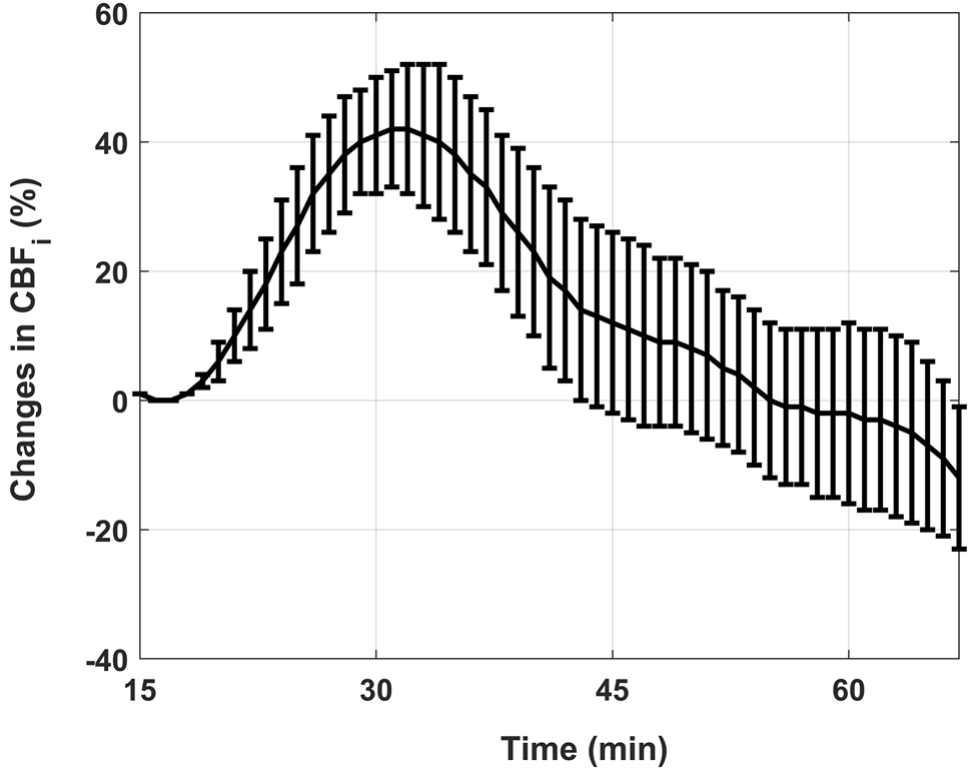
Changes in CBFi obtained by averaging the ΔCBFi(t) curves from all subjects at each time point. The bars are the standard errors and the temporal axis was computed by taking the average time of the shifted ΔCBFi(t) curves as illustrated in Fig. 3b.

Figure 4 displays the time-dependent changes in ${CBF}_{i}$ obtained by computing the mean, at each time point, of the temporally shifted $\Delta CBF_{i} \left( t \right)$ curves of all subjects. The x-axis in Fig. 4 is the mean time obtained after temporal shifting, as illustrated in Fig. 3b. Note that the ${CBF}_{i}$ data from the first subject was removed since our initial protocol was to stop after 60 min of DCS acquisition. This subject required a second dose of insulin − to achieve clinically relevant hypoglycemia—which was given 14 min into the DCS acquisition and by the 60 min mark, when we stopped the acquisition, ${CBF}_{i}$ was still rising. Since the $\Delta {CBF}_{i}$ data from this individual did not include the decay phase that was noticed in all the other subjects, we excluded the data from this individual to avoid biasing the $\Delta {CBF}_{i}$ results.

The middle column of Table 1 displays the time between the insulin bolus and the first appearance of neurogenic hypoglycemia symptoms. The last column represents the time between the insulin challenge and the initial rise of ${CBF}_{i}$. As expected, there was variability between individuals; however, ${CBF}_{i}$ started to increase before the appearance of neurogenic symptoms in 5 out of the 7 subjects. Notably, the mean delay between insulin injection and the rise of ${CBF}_{i}$ was shorter than the delay between the appearance of hypoglycemia symptoms and the insulin bolus; however, the difference was not statistically significant.

**Table 1:** Delay between insulin injection, hypoglycemia symptoms, and ${CBF}_{i}$ rise.

Subject #	Insulin bolus to symptoms (min)	Insulin bolus to ${CBF}_{i}$ rise (min)
2	30	33
3	30	24
4	35	3
5	12	5
6	9*	18*
7	35	20
8	20	16
Mean (SD)	24 (11)	17 (10)

Patient who received a 2nd dose of insulin.

*Time of 2nd insulin bolus was used as reference.

Table 2 displays the blood glucose and cortisol levels measured at the rise of ${CBF}_{i}$. Since the BG and cortisol level were only sampled every 15–30 min, the data in Table 2 represent the measurements acquired at the time point that was the closest to the rise of ${CBF}_{i}$. Note that in all subjects, ${CBF}_{i}$ started to rise before the cortisol level reached its maximum value.

**Table 2:** Blood glucose and cortisol levels at the rise of the cerebral blood flow index.

Subject #	Blood glucose (mM)	Cortisol (nM)
1	2.4	162
2	1.3	312
3	1.3	177
4	1.6	245
5	2.1	212
6	1.5	104
7	1.7	404
8	1.3	513
Mean (SD)	1.7 (0.4)	266 (137)

**Figure 5:**
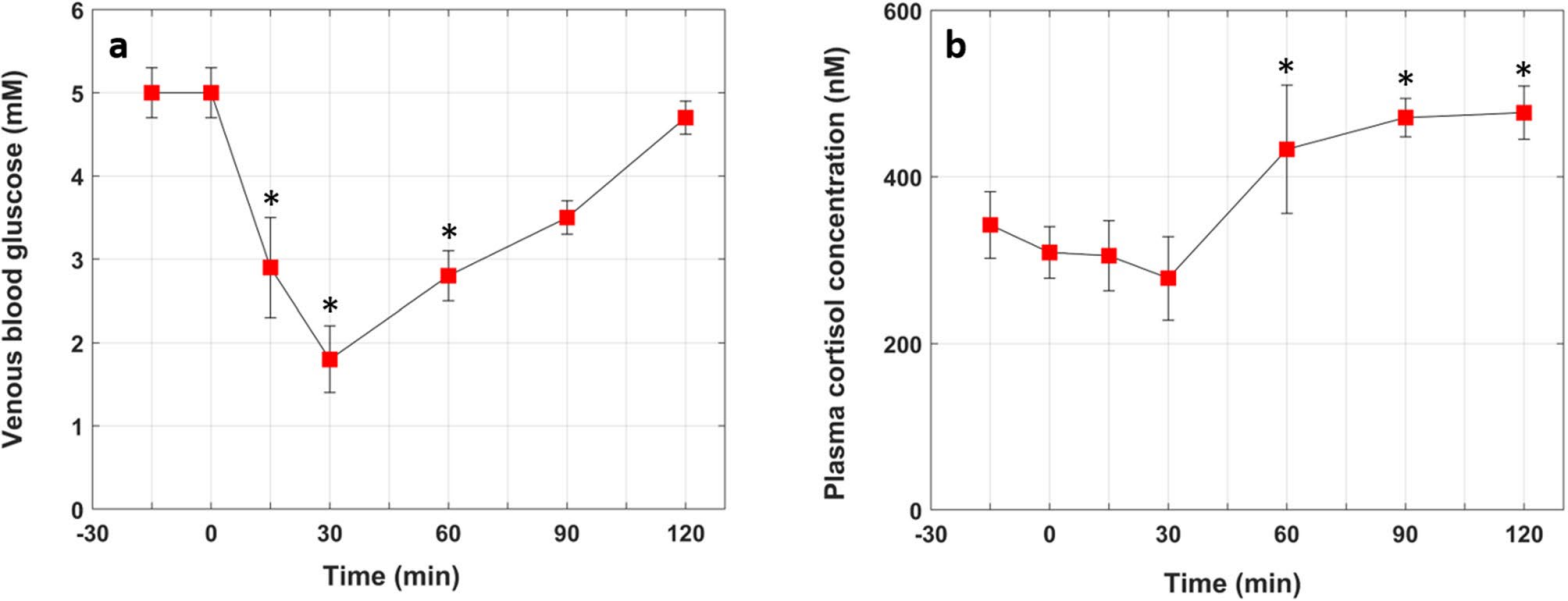
Plasma blood glucose (**a**) and plasma cortisol (**b**) concentrations measured in all subjects. The red squares are the mean values and the error bars represent the standard errors. * In (**a**) indicate blood glucose values that are significantly different from the baseline value at -15 min while the * in (**b**) indicate cortisol values that are significantly different from the lowest cortisol level (measured at the 30 min mark).

Figure 5 shows the concentrations of glucose and cortisol in the blood for all subjects during the entire study, starting 15 min before the insulin bolus and ending 2 h after the insulin injection. There was a statistically significant effect of time on both blood glucose (F(6,42) = 14.098, *p* < 0.05) and cortisol levels (F(6,42) = 6.029, *p* < 0.05). For both blood glucose and cortisol measurements, a post-hoc Tukey test revealed significant differences between baseline and after insulin injection at the *p* < 0.05 level (Fig. 5). Effect sizes of partial η^2^ = 0.466 (cortisol) and partial η^2^ = 0.672 (blood glucose) were used to determine that these statistically significant effects were measured at a power greater than 0.8. Two hours after the insulin bolus, the blood glucose returned to baseline while the cortisol level remained above the baseline concentration, in agreement with the findings of Teh et al.^31^.

## Discussion

This is the first report of *dynamic* response of cerebral blood flow (CBF) to hypoglycemia in humans. As such, it is an important contribution to the understanding of the effects of acute hypoglycemia on human cerebral perfusion. Since abnormal changes in CBF are involved in the pathogenesis of hypoglycemia unawareness^11,41,42^, the ability to monitor the dynamics of CBF during acute hypoglycemia could provide new insights into the pathogenesis of this serious condition. Further, it is important to state that although we refer to the changes in the diffusion coefficient, computed using Eq. (4), as changes in cerebral blood flow index, many studies have demonstrated that these changes reflect changes in cerebral perfusion.

An intriguing finding of the current study is that the onset of ${CBF}_{i}$ increment may precede the appearance of neurogenic symptoms. Table 1 shows that despite the heterogeneity in the temporal response of the cerebral perfusion (i.e., the delay between the insulin bolus and the rise of ${CBF}_{i}$), on average ${CBF}_{i}$ started to increase 7 min before the appearance of hypoglycemia symptoms. Increased CBF in response to insulin-induced hypoglycemia is well documented in the literature but previous human studies were conducted under steady-state conditions using glycemic clamp^8,10,13^. Therefore, it was not possible for these studies to document the temporal relationship between CBF rise and onset of neurogenic symptoms. The present findings suggest that the CBF response to hypoglycemia may precede the appearance of neurogenic symptoms in nondiabetic subjects with normal awareness of hypoglycemia. This is in agreement with Page et al. who, using arterial spin labeling MRI, found that neuronal activity precedes the rise in systemic counterregulatory hormones during hypoglycemia, suggesting that brain activation may precede hypoglycemia symptoms; however, they did not report patient symptoms^43^. Nevertheless, the findings of both studies need to be confirmed with future investigations with larger sample sizes. Notably, the current study has a power of 0.24 (effect size of 0.56); thus, a sample size of 28 subjects would have been required to detect a statistically significant difference with a power of 0.8.

Cerebral blood flow index started to rise 17 min after insulin injection and reached its maximum at the 32 min mark with an average increase of 42%, compared to the baseline ${CBF}_{i}$*.* Interestingly, ${CBF}_{i}$ reached its maximum when the blood glucose was at its nadir (1.8 ± 04 mM). Presumably, blood flow increased in the frontal cortex, as a protective compensatory mechanism, to enhance the supply of glucose to the brain^44^. These results agree with the physiological model of counterregulatory hypoglycemia responses, including increased cerebral perfusion to enhance glucose delivery to the brain, to restore euglycemia^10^. This is not surprising since the brain is one of the most metabolically active organs, glucose is its principal metabolic fuel, and endogenous cerebral levels of glucose are low; thus, the brain relies on continuous supply of glucose from the blood to maintain normal metabolic functions and any prolong disruption in glucose delivery can be devastating^6,7^. The brain has developed sophisticated regulatory mechanisms to preserve its glucose supply^45^ and one of such mechanisms is to increase cerebral perfusion to enhance glucose delivery to the brain in response to hypoglycemia.

CBF increments in response to insulin-induced hypoglycemia may be mediated by adenosine and ATP-sensitive potassium channels (KATP). Notably, Horinaka et al.^12^ showed that in rodents, caffeine ‒ an adenosine receptor antagonist ‒ reduced the CBF response to insulin-induced hypoglycemia in a dose-dependent manner with complete elimination at a dose of 20 mg/kg. They also found that like caffeine, the oral hypoglycemic diabetic medication glibenclamide—a KATP channel inhibitor—produced dose-dependent reductions of the CBF response during hypoglycemia; CBF increases were 66% in control rats, 25% with 1 µM of glibenclamide, and almost complete blockade (5%) with 2 µM of glibenclamide. Furthermore, a direct effect of insulin or nitric oxide on CBF is unlikely since it has been shown that insulin administered under euglycemic conditions had no effects on CBF^11,46^. As well, chronic blockade of nitric oxide synthase activity in conscious rats did not significantly reduce the CBF response to insulin-induced hypoglycemia^46^.

Furthermore, the $\Delta {CBF}_{i}(t)$ increments are followed by a sharp decrease (Fig. 4). This could be caused by decreased cerebral metabolic rate of glucose (CMRgl) since it has been shown that CMRgl decreases when blood glucose levels fall below 2.5–3.3 mM^47^. The decrease in $\Delta {CBF}_{i}(t)$ could also be caused by increased blood glucose since, as shown by Fig. 5a, blood glucose started to rise after the 30 min mark. However, the fact that $\Delta {CBF}_{i}(t)$ continued to decrease despite the rise in blood glucose after the 30 min mark, suggest that decreased CMRgl is likely the major driver of the $\Delta {CBF}_{i}(t)$ decrease. Notably, the sharp decrease in $\Delta {CBF}_{i}(t)$ noticed in Fig. 4 is in contrast with the sustained high CBF, even after normalization of counter-regulatory hormones and hypoglycemia symptoms, reported in healthy subjects who underwent a 66 min hypoglycemia clamp^48^. This further points to the difference between dynamic hypoglycemia and the widely used glycemia clamp approach.

The current study has some limitations. The numbers of subjects were small in keeping with a pilot study; none of the subjects had diabetes; a single-source detector pair was used which did not allow assessment of changes in skin perfusion. Using additional source-detector pairs, including one with a short source-detector separation, would enable us to assess changes in cutaneous blood flow since it has been shown that in nondiabetic subjects, cutaneous blood flow − measured with the laser Doppler technique − increased in the forehead at blood glucose of 1.8 mM^49^. Furthermore, the 42% increase in $\Delta CBF_{i} \left( t \right)$ measured in the current study is larger than the typical CBF increase reported by previous studies with PET and MRI − even although, in a study using pulsed arterial spin labeling MRI, Page et al. reported a twofold increase in hypothalamic perfusion during hypoglycemia when compared to euglycemia (44.5 ml/100 g/min vs 22.0 ml/100 g/min)^43^. It should be noted that previous reports used hypoglycemic clamp with BG levels typically maintained above 2 mM, while in the current study BG levels dropped below 2 mM in all subjects. Since CBF increases significantly only when BG level drops to 2 mM or lower^9^, the difference in BG level could explain the relatively larger increase in ${CBF}_{i}$ reported herein. Further, due to technical challenges, we were not able to monitor tissue optical properties during the ${CBF}_{i}$ measurements. Though it is unlikely that tissue scattering changes significantly from euglycemia to hypoglycemia, light absorption could change between the two conditions. Continuous monitoring of tissue optical properties would enable us to account for the potential confounding effects of hypoglycemia-induced vasodilation and the ensuing increase in tissue absorption. Vasodilation could increase both cerebral and scalp blood content. Not accounting for this confounder would results in overestimation of the ${CBF}_{i}$ increments, which could explain the larger increase in $\Delta {CBF}_{i}(t)$ reported in the current study. Future studies should include a method that accounts for potential hypoglycemia-induced changes in skin blood flow and tissue absorption by using a multi-distance hybrid optical device that combines near-infrared spectroscopy and DCS^50^. Furthermore, given the expected heterogeneity of the ${CBF}_{i}$ response to insulin, it was necessary to shift the $\Delta CBF_{i} \left( t \right)$ curves to align the onset of their rises. The effect of the shifting is to smooth some features of the individual curves such as sharp rise and fall, similar to applying a low pass filter to the data. We choose the initial rise of the $\Delta CBF_{i} \left( t \right)$ curves as reference but could choose to align the curves so that their maxima coincide. The choice of the reference point will slightly change the smoothing and, consequently, the shape of the averaged $\Delta CBF_{i} \left( t \right)$ curve. However, the general finding of increased ${CBF}_{i}$ due to hypoglycemia will be maintained as well as the finding that ${CBF}_{i}$ increase precede hypoglycemia symptoms. Another limitation of the study is the low blood glucose sampling rate, as the subjects followed the normal clinical protocol of plasma glucose sampling no more often than every 15 min. More frequent sampling of glucose may have allowed for more precise associations between CBF and blood glucose. Future studies could address this limitation by measuring blood glucose more frequently with continuous subcutaneous glucose monitoring.

In summary, changes in cerebral blood flow index was measured with diffuse correlation spectroscopy (DCS) during insulin-induced acute hypoglycemia in nondiabetic subjects with normal awareness of hypoglycemia. We show that DCS can continuously monitor $\Delta CBF_{i} \left( t \right)$ in adult subjects during hypoglycemia and, more importantly, the onset of ${CBF}_{i}$ increases seems to precede the appearance of hypoglycemia symptoms. However, due to the low temporal resolution of the plasma glucose sampling, it was not possible to compare the $\Delta CBF_{i} \left( t \right)$ values with the BG data. Future work should address this limitation by sampling the BG more frequently to assess the dynamic coupling of CBF and BG. Given that DCS is safe, portable, and can monitor ${CBF}_{i}$ with high temporal resolution, it could open new opportunities to elucidate the dynamic relationship between CBF and hypoglycemia, and to provide new insights into the pathogenesis of hypoglycemia unawareness.
